# Reduced Motor Individuality in Older Adults Revealed by Network-Based Gait Fingerprinting

**DOI:** 10.3390/medicina61081454

**Published:** 2025-08-12

**Authors:** Emahnuel Troisi Lopez, Roberta Minino, Mariam Maisuradze, Francesca Latino, Maria Giovanna Tafuri

**Affiliations:** 1 Department of Education and Sport Sciences, Pegaso University, 80143 Naples, Italy; 2 Department of Medical, Motor and Wellness Sciences, University of Naples “Parthenope”, 80133 Napoli, Italy; 3 Department of Literary, Linguistic and Philosophical Studies, Pegaso University, 80143 Naples, Italy

**Keywords:** motor control, gait analysis, neurodegeneration

## Abstract

*Background and Objectives*: Gait is a fundamental human behavior essential for individual autonomy and well-being; it reflects a complex inter-joint coordination that can change with aging. *Materials and Methods*: This study applied a network-based fingerprinting approach to evaluate the stability and individuality of gait coordination in adults (mean age: 41.6) and older adults (mean age: 73.5). Each participant completed two gait recordings, from which we constructed kinematic networks (i.e., kinectome) representing joint–velocity correlations. Then, borrowing from network fingerprinting techniques, we computed measures of intra-subject similarity (Iself), inter-subject similarity within the same group (Iothers), cross-group similarity (Iextra), and individual discriminability (Differentiation rate, DR). *Results*: While Iself was comparable across groups, older adults showed higher Iothers and lower DR, indicating more homogeneous and less distinctive coordination patterns. Furthermore, Iothers was significantly higher than Iextra in the older group only, suggesting age-specific convergence in motor behavior. *Conclusions*: These findings support the hypothesis that aging reduces the individuality of gait coordination, possibly due to adaptive or degenerative changes in motor control. Kinectome-based fingerprinting thus offers a promising tool for capturing subtle shifts in neuromotor organizations linked to aging.

## 1. Introduction

Human gait is characterized by highly coordinated motor behavior, which reflects the integrity of the neuromuscular system and cognitive–motor behavior. It is well known that aging can impact gait performance, alter balance, increase variability, and determine a higher risk of falls in older adults [1,2,3]. Gait disorders may arise from multiple causes, including neurodegenerative diseases such as Parkinson’s and Alzheimer’s disease, cerebrovascular events such as stroke, musculoskeletal impairments involving joints and muscles, and age-related decline in sensory and proprioceptive functions. Furthermore, cardiovascular conditions, medication side effects, and psychological factors such as fear of falling can also contribute to impaired gait performance [4]. To detect and evaluate these disorders, several diagnostic methods have been developed. Traditional clinical assessments often include observational gait analysis, functional mobility scales, and standardized tests, such as the Timed Up and Go (TUG) or the 10-Meter Walk Test [5]. In addition, instrumental approaches, such as motion capture systems, wearable inertial measurement units, and force platforms, provide quantitative measures of spatiotemporal parameters, kinematic patterns, and balance control, allowing for more precise evaluation of gait alterations [6,7,8]. Studies on aging and related gait changes have primarily focused on isolated spatiotemporal and variability-related metrics (e.g., walking speed, stride length, step variability), offering useful but limited insight into coordination [9]. While these variables capture certain aspects of motor decline, they often fail to encompass the dynamic interactions among joints and locomotion upon which coordination is built. Indeed, aging is associated with modifications in motor control, including reduced proprioception, altered intermuscular synergies, and increased co-activation of muscles related to joints, such as the hip, knee and ankle, which are mainly employed during walking [10,11]. This complex framework, represented by a fine tuning of many joints and many degrees of freedom under the material (e.g., ligaments, tendons) and intangible conceptual factors (e.g., space and time) suggest that more sophisticated analytical frameworks able to represent inter-joint coupling and whole-body coordination may be necessary to fully understand age-related alterations in gait function.

Understanding the mechanisms of coordination during gait can be facilitated by interpreting the kinematics of the locomotion as a network, where joints interact dynamically and generate movement patterns. Traditional methods often analyze coordination between only a few joints or use phase-based metrics that cannot represent holistic inter-articular relationships [12,13]. To address this limitation, recent studies have applied network science to kinematic data, modeling joints as nodes and their temporal interactions as edges defined by correlation metrics (e.g., Pearson coefficients). This framework reveals the structural topology of locomotor coordination [13], which can help understanding related to the effect of aging on coordination.

In previous studies, we enclosed this information into a matrix-shaped mathematical object named the kinectome, which constructs a comprehensive kinematic network of human movement. According to this approach, nodes correspond to anatomical landmarks (e.g., joint centers) and edges represent the Pearson correlation of their kinematic time series, such as accelerations or angular velocities [14,15]. This network-based representation not only encapsulates inter-joint synchronization across gait cycles but can also be analyzed using graph-theoretical tools to assess modularity, symmetry, and fingerprinting capabilities [14,15,16]. To this regard, the kinectome has shown its utility in both healthy individuals and patients with Parkinson’s disease, revealing alteration in whole-body coordination and reduced symmetry. Furthermore, it allows individua’ differentiation based on short gait recordings, through a methodological approach realized using network-based fingerprinting [17,18]. Originally developed in the field of brain connectomics [19], fingerprinting techniques have recently been extended to the domain of human movement, where they enable the assessment of individual specificity and temporal stability of kinematic networks. In this context, each subject’s motion is represented by a specific pattern (i.e., the kinectome). By comparing these patterns across multiple recordings and across different individuals, it becomes possible to investigate the reproducibility and distinctiveness of a subject’s motor behavior. Building on this network perspective of gait, it becomes possible not only to describe coordination patterns but also to quantify how uniquely these patterns identify individuals or groups. To do so, similarity indices are computed between each subject’s kinectome and that of other recordings. The degree of self-similarity, known as Iself, reflects how consistently a subject’s coordination pattern is preserved across time. Conversely, the average similarity between a subject and the other individuals within the same group, referred to as Iothers, provides a measure of how homogeneous or heterogeneous the motor patterns are among individuals of the same group [20]. From these two metrics, it is possible to derive a discriminative measure, often referred to as the Differentiation Rate (DR), which quantifies how often a subject is more like themselves than they are to others, hence calculating the chance of recognizing the motor patterns belonging to the same individual [21]. Finally, by computing the similarity between a subject belonging to different groups (Iextra), one can assess the extent to which gait coordination patterns are different between populations [22].

By the means of fingerprinting analysis, it is possible to capture intra-individual stability, inter-individual variability, and between-group separability within a unified network-based approach. We believe that this double level analysis (i.e., subject-level and group-level) may reveal individual motor signatures, offering insight into how aging may influence not only the stability of movement, but also its individuality. Aging is known to affect the adaptability and variability of motor behavior [23,24], with several studies suggesting that older adults often adopt more stereotyped and constrained motor patterns as a compensatory response to declining sensorimotor resources [25]. While such adaptations may support functional mobility, they may also reduce the heterogeneity and uniqueness of movement, leading to greater intra-group similarity within older populations. This phenomenon remains underexplored from the point of view of coordination. Previous studies focus on group-level averages or single-joint metrics, leaving open the question *“how does aging affect the individual structure of coordination during walking?”* Exploiting the kinectome (i.e., network-based coordination assessment) and fingerprinting metrics, it becomes possible to analyze gait patterns at individual levels and in age-grouped populations, investigating how distinguishable their coordination signatures are within and across age-defined populations.

In this study, we addressed this question by analyzing gait data from healthy adults and older adults, each recorded twice during the same session. We applied a Kinectome-based fingerprinting approach to quantify intra-individual stability, inter-individual similarity, and between-group separability of lower-limb coordination patterns. Our aim was to determine whether aging is associated with reduced individuality and increased homogeneity in gait coordination, as revealed by the structure and reproducibility of kinematic networks.

## 2. Methods

### 2.1. Study Design

This study is a cross-sectional observational comparative investigation aimed at examining differences in motor coordination patterns during gait between two groups: adults and older adults. Participants, selected according to clear inclusion and exclusion criteria, underwent kinematic gait assessment using a stereophotogrammetric system. Multiple walking trials were recorded for each subject, from which similarity and distinctiveness indices of motor patterns were derived through correlation and fingerprinting analyses.

**Figure 1:**
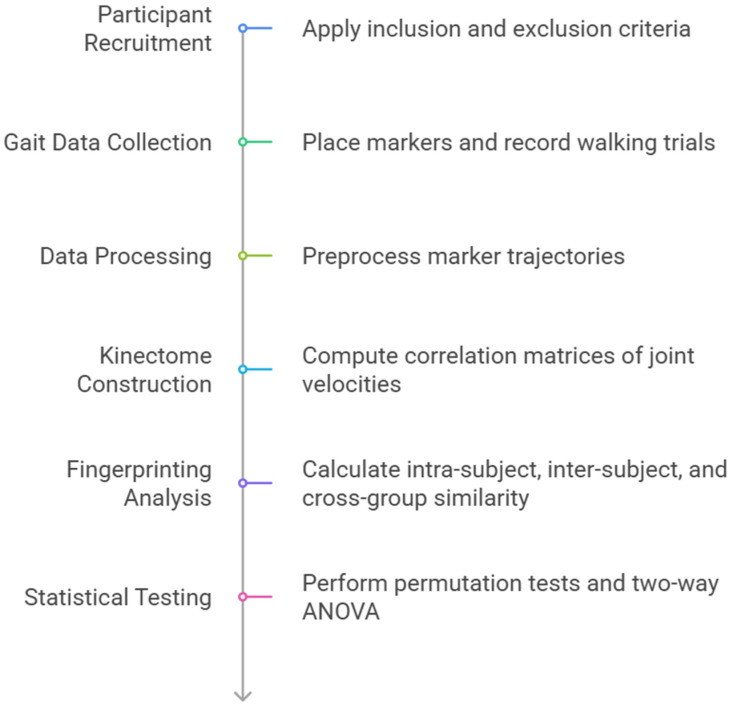
Study flowchart.

The following figure illustrates the study flowchart (Figure 1).

### 2.2. Participants

Twenty-six individuals participated in the study, including 12 adults (mean age: 41.6 ± 6.4 years; 2 females) and 14 older adults (mean age: 73.5 ± 3.5 years; 4 females). Inclusion criteria were age within the specified adult or older adult ranges, community-dwelling status, ability to walk independently without the use of assistive devices, and absence of neurological, musculoskeletal, or cognitive impairments potentially affecting gait. Particular attention was given to excluding chronic cerebrovascular conditions, such as chronic cerebral ischemia, through a combination of medical history screening, self-report questionnaires, and caregiver information when applicable. Participants with a previous diagnosis of cerebrovascular disease, transient ischemic attacks, or stroke, as well as those under pharmacological treatment for chronic cerebral ischemia, were not eligible. Although no additional neuroimaging or formal neurological evaluations were performed within the study protocol, available medical documentation, including prior neuroimaging reports and specialist diagnoses, was carefully reviewed to minimize the risk of including individuals with undiagnosed cerebrovascular pathology. None of the participants had consumed alcohol within the 48 h preceding data collection. Exclusion criteria comprised recent lower-limb injuries (within the past six months), history of orthopedic surgery, alcohol abuse, and any diagnosed conditions known to impair balance or locomotion. To provide a comprehensive overview of the sample and to address potential confounding factors influencing gait, the main demographic, anthropometric, and clinical characteristics of the participants are summarized in Table 1.

In order to ensure the adequacy of the sample, a priori power analysis was conducted using G*Power 3.1 (Heinrich-Heine-Universität Düsseldorf, Düsseldorf, Germany). Based on previous literature on gait biomechanics in aging populations, we assumed a medium effect size (f = 0.25), an alpha level of 0.05, and a statistical power of 0.80 for repeated measures ANOVA. The analysis indicated a minimum required sample size of 24 participants. Our final sample, consisting of 26 individuals (20 males and 6 females), met this threshold, thereby providing sufficient statistical power to detect the reported effects.

Participants provided written informed consent prior to participation. The study was approved by the local ethics committee and conducted in accordance with the Declaration of Helsinki.

### 2.3. Gait Data Acquisition

Gait kinematics were collected using a Qualisys stereophotogrammetric system equipped with eight cameras operating at 120 Hz (ProReflex Unit-Qualisys Inc., Gothenburg, Sweden). Fifty-five passive reflective markers were placed on anatomical landmarks according to a modified Davis protocol [26,27]. Participants performed four walking trials along a straight 10-m path at their self-selected speed. For each subject, the two cleanest trials were selected for analysis, based on signal quality and absence of artifacts. Each trial was segmented into a double gait cycle, beginning with a heel strike of one foot and ending with the second heel strike of the contralateral foot, as in Lopez et al. 2022 [28]. This allowed us to include a complete gait cycle for each leg. Time series of the markers were preprocessed in Visual3D (Professional v6.02, C-Motion, Inc., Germantown, MD, USA) using an automated pipeline that included gap interpolation and low-pass filtering to reduce noise. The software also calculated the time series of joint angles during the gait cycle, which were then used for the subsequent analysis.

### 2.4. Kinectome Construction

**Figure 2:**
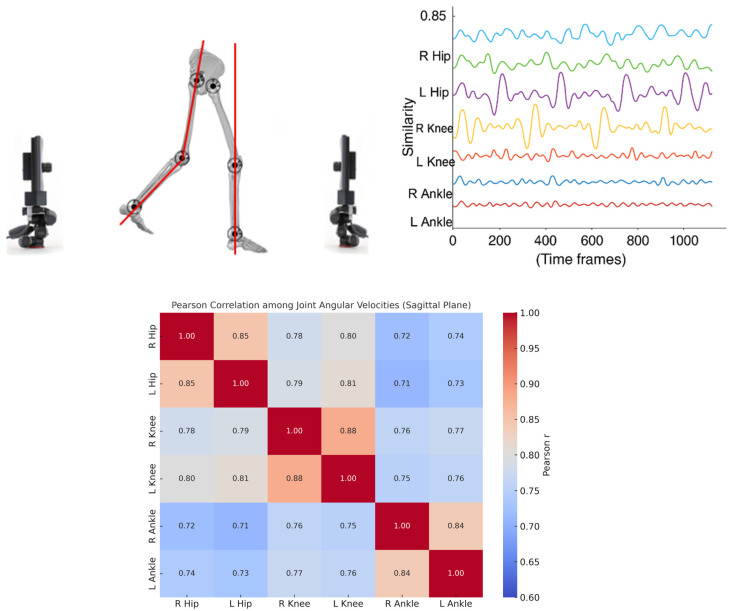
Analysis of lower-limb joint coordination during gait. The left panel shows a schematic representation of joint angles in the sagittal plane while walking. The top-right panel illustrates time series of joint similarity values (e.g., angular velocity patterns) for the right and left hips, knees, and ankles across time frames. The bottom-right panel presents a Pearson correlation matrix quantifying the similarity among joint angular velocities, indicating strong intra-limb and inter-limb coordination, particularly between homologous joints (e.g., left and right hips, knees, and ankles).

For each trial, a kinematic network (i.e., kinectome) was computed based on the sagittal-plane velocities of six lower-limb joints: left and right ankle, knee, and hip. Pearson correlation coefficients [29] were calculated between each pair of joint–velocity signals, resulting in a 6 × 6 symmetric correlation matrix for each trial. This matrix represented the subject-specific coordination structure for that recording. The analysis focused on the sagittal plane, as this is the primary plane in which joint excursions during gait predominantly occur [30] (Figure 2).

### 2.5. Fingerprinting Analysis

For each subject, the two kinectomes obtained from separate trials were compared to compute a set of fingerprinting metrics [17]. Intra-subject similarity (Iself) was defined as the Pearson correlation between the subject’s own kinectomes. Inter-subject similarity (Iothers) was computed as the average correlation between the subject’s trials and the trials of all other subjects within the same group. Discriminability (Differentiation Rate, DR) was calculated as the percentage of Iothers values that were lower than the subject’s own Iself, indicating the subject’s ability to be differentiated within the group. Cross-group similarity (Iextra) was measured as the average similarity between a subject and the members of the opposite age group.

### 2.6. Statistical Analysis

All statistical analyses were performed in MATLAB R2020b (MathWorks, Inc., Natick, MA, USA). Permutation tests (10,000 iterations) based on the means were used to assess group differences in Iself, Iothers, and DR, with the null distribution generated by randomly shuffling group labels [31]. A two-way ANOVA [32] was conducted using the anovan function to evaluate the effects of group (adult vs. older adult) and similarity condition (Iothers vs. Iextra) on correlation values. Boxplots were used to visualize group-level distributions of each fingerprinting metric. Significance was set at *p*-value < 0.05.

## 3. Results

**Figure 3:**
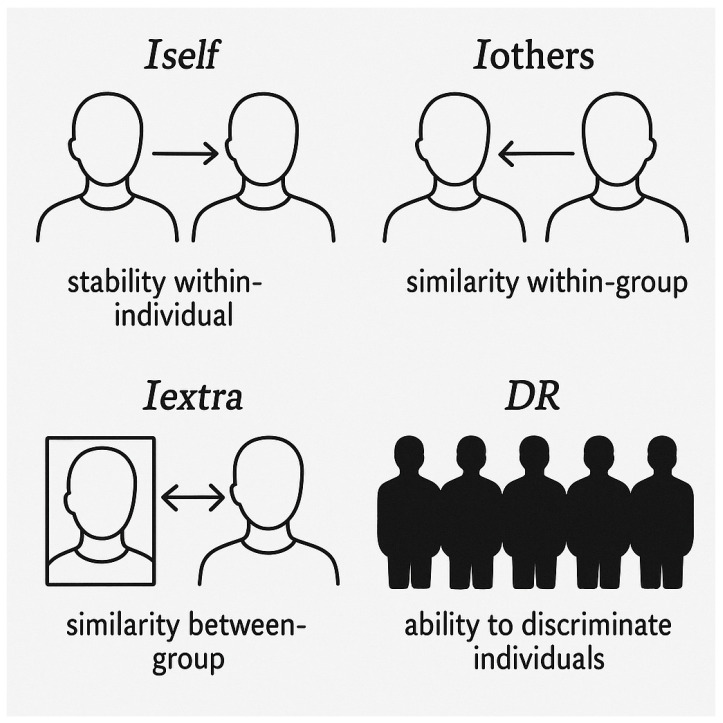
Schematic representation of the four indices used to analyze identity and individual discriminability. *Iself* indicates within-individual stability over time; *Iothers* represents similarity between individuals within the same group (within-group similarity); *Iextra* shows similarity between individuals from different groups (between-group similarity); *DR* (Discrimination Ratio) highlights the ability to distinguish one individual from others, reflecting their uniqueness within the sample.

All participants (12 adults and 14 older adults) completed two valid gait recordings in a single session. For each subject, a kinectome was constructed from each recording, and fingerprinting metrics were computed to evaluate intra-individual stability (Iself), inter-individual similarity within the same group (Iothers), discriminability (DR), and similarity across groups (Iextra), using Pearson correlation (Figure 3).

**Figure 4:**
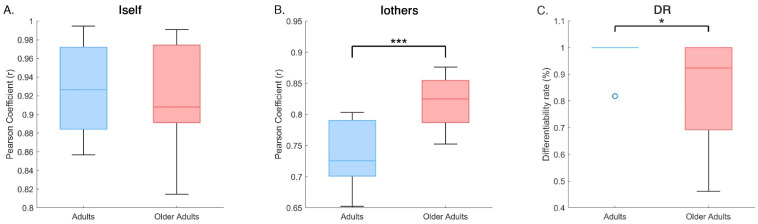
Group comparisons of gait fingerprinting metrics. Box plots show the distribution of four main fingerprinting measures for adults and older adults: (**A**) Iself, intra-subject similarity; (**B**) Iothers—average similarity to other members of the same group; (**C**) DR—Differentiation Rate, reflecting the proportion of comparisons in which Iself exceeded Iothers. Statistical significance was assessed using permutation tests (10,000 iterations). Asterisks indicate significant differences between groups (* *p* < 0.05, *** *p* < 0.001).

Permutation tests (10,000 iterations) revealed no significant difference in Iself between groups (adults: 0.93 ± 0.05; older adults: 0.92 ± 0.06; *p* = 0.628), indicating comparable intra-subject stability across age. However, Iothers was significantly lower (*p* < 0.001) in adults (0.74 ± 0.05) than in older adults (0.82 ± 0.04), suggesting greater heterogeneity in coordination patterns among the adult group. This difference was found also in DR values, with adults showing a significantly higher (*p* = 0.04) differentiability rate (0.97 ± 0.07) compared to older adults (0.85 ± 0.19), indicating a reduced ability to distinguish individual gait patterns within the older population. These results are shown in Figure 4.

**Figure 5:**
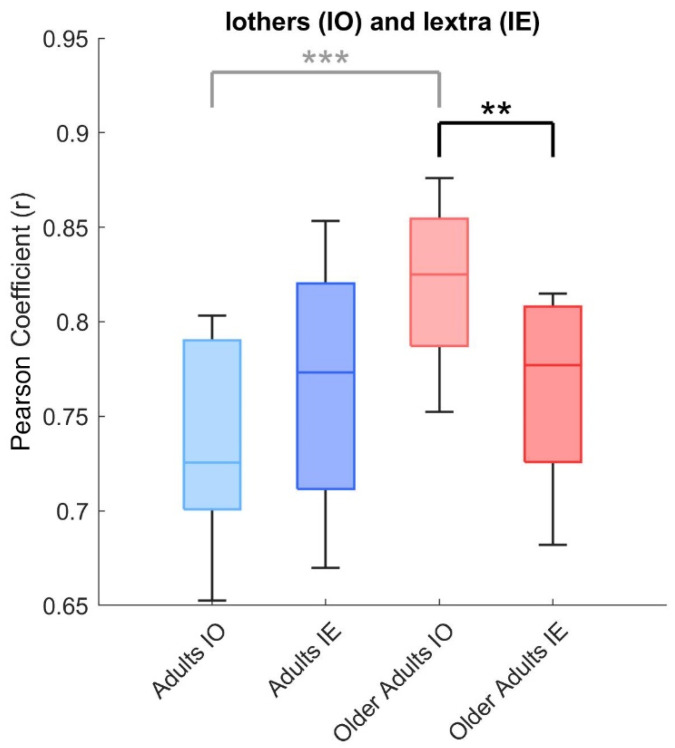
Interaction between group and condition in similar metrics. Box plots show the comparison between intra-group similarity (Iothers) and cross-group similarity (Iextra) for both adults and older adults. A two-way ANOVA revealed significant main effects of group and group × condition interaction. Post hoc tests indicated that, in older adults, Iothers was significantly higher than Iextra, whereas no such difference was observed in the adult group. Difference between both group is reported in light grey, as shown in Figure 1, yet. Asterisks indicate significant differences between groups (** *p* < 0.01, *** *p* < 0.001).

A two-way ANOVA on Iothers and Iextra, with group (adults, older adults) and condition (intra-group, cross-group) as factors, revealed significant main effects of group (F(1, 48) = 8.45, *p* = 0.006) and interaction (F(1, 48) = 8.45, *p* = 0.006). As shown in Figure 5, post hoc comparisons showed that, in older adults, Iothers was significantly higher than Iextra (0.82 ± 0.04 vs. 0.77 ± 0.05; *p* = 0.003), indicating that older adults were more similar to peers in their own group than to adults in the other group. No such difference was observed in the adult group (Iothers: 0.74 ± 0.05; Iextra: 0.77 ± 0.06; *p* = 0.233).

In addition to statistical significance, effect sizes were calculated to provide a more accurate interpretation of the magnitude of the observed differences. For intra-subject stability (Iself), the analysis revealed a Cohen’s d of 0.18, indicating a very small effect, consistent with the absence of significant differences between adults and older adults. In contrast, inter-subject similarity (Iothers) showed a very large effect (Cohen’s d = −1.78), suggesting greater heterogeneity in coordination patterns within the adult group compared to the older group. Similarly, the differentiability rate (DR) displayed a large effect (Cohen’s d = 0.81), confirming a higher ability to discriminate individual gait patterns in the adult group. Finally, the two-way ANOVA yielded an eta squared (η^2^) of 0.15, which represents a large effect, further strengthening the robustness of the observed differences in relation to the main effect of group and the group × condition interaction.

These results (Table 2) confirm that, although intra-subject stability does not substantially differ between the two cohorts, coordination structure and the ability to differentiate individual gait patterns are significantly modulated by age, with large effect sizes supporting the reported group differences.

Together, these results indicate that, while both age groups maintain stable individual gait coordination across repeated recordings, older adults tend to walk in more similar ways to one another and exhibit reduced motor individuality compared to adults.

## 4. Discussion

Our study aimed at exploring how aging influences the stability and individuality of gait coordination patterns by applying a network-based fingerprinting approach to adult and older adult populations. Our findings showed both common and different aspects between the two groups: while intra-subject stability of gait coordination (i.e., the Iself) remained comparable between adults and older adults, differences between the two groups emerged in inter-subject similarity and differentiability. Specifically, older adults exhibited significantly higher inter-individual similarity (Iothers) and lower discriminability, suggesting that their gait patterns were more homogeneous and less uniquely identifiable than those of adults. Moreover, we found that, exclusively in the older group, the similarity between individuals within the group (Iothers) was significantly greater than the similarity with individuals from the other group (Iextra).

These results suggest that, although the ability to reproduce one’s own coordination pattern remains relatively intact with age, the individual characteristics of gait are reduced in older adults. In contrast, adults displayed more idiosyncratic coordination patterns, with lower similarity within the group and higher self-similarity. This dual observation (i.e., preserved internal stability but reduced external distinctiveness) raises important questions about how aging affect motor characteristics in a way that alters the balance between functional consistency and behavioral subjectivity. The reduction in inter-individual variability and distinctiveness of gait patterns among older adults aligns with theoretical models of motor aging, suggesting a loss of adaptability and complexity in motor control systems [33]. According to Newell’s constraints model, motor behavior stems from a dynamic interaction that involves the individual, the task, and the environmental constraints [23]. As an individual ages, the degrees of freedom available for motor coordination may become increasingly limited due to neuromuscular, sensory, and cognitive decline, which can reduce the availability of coordination strategies [34]. This restriction may lead older adults to modify their gait pattern towards stereotyped motor solutions, looking for strategies that are functionally sufficient but less flexible or individualized. Such modification may explain the high Iothers scores observed in our older group, suggesting that age-related changes may not necessarily impair the internal consistency of coordination but rather reduce the heterogeneity of available movement patterns. This interpretation is supported by previous studies showing increased joint stiffness, greater muscle co-contraction, and diminished variability in motor output among older individuals [24,25,35]. However, it should be stated that, while these adaptations may promote stability and reduce fall risk, they also bring reduced motor richness and individuality. Similarly, these findings are also in line with the well-known “loss of complexity” in aging, whereby physiological systems become less adaptable and more predictable over time [36]. Within the movement and gait framework, this may arise through more uniform coordination strategies across individuals and a diminished capacity to express unique motor signatures. Thus, the reduced discriminability in older adults observed in our fingerprinting analysis may reflect an age-related shift from flexible, self-organizing motor behavior to more rigid, homogenized patterns driven by biological constraints. Furthermore, several studies reported that aging is related to increased muscle co-contraction in ankle and knee joints, which contributes to joint stiffness and simplified motor output [35,37]. This heightened co-activation may serve to stabilize posture and enhance safety during walking but can inadvertently reduce the degrees of freedom available for coordination, aligning with our observation of more homogeneous kinectome patterns in the older group. It is also important to note that chronic cerebrovascular conditions, such as chronic cerebral ischemia, which are known to negatively impact gait performance in the elderly [38], were carefully excluded through detailed anamnesis and medical history screening. Nonetheless, we cannot completely rule out the presence of subclinical forms that are not detectable without advanced imaging, which may represent a residual confounding factor.

It should also be considered that our sample included a gender imbalance, with 20 males and only 6 females. This aspect deserves attention because several studies have documented sex-related differences in gait biomechanics, including pelvic motion, stride length, cadence, and joint coordination strategies [39,40,41]. Women, for instance, often exhibit greater pelvic obliquity and different muscle activation patterns compared to men, which may influence coordination dynamics and network-based measures, such as Iothers and discriminability [42]. Although our analyses did not reveal explicit sex-specific effects, the limited number of female participants reduces our ability to determine whether the observed age-related trends apply similarly across sexes.

Together, this converging evidence strengthens the interpretation that age-related biomechanical adaptations do not just affect isolated variables, but affect the whole coordination network of gait, reducing both individual uniqueness and group heterogeneity, which is instead observed in healthy adults [43].

Several limitations should be acknowledged when interpreting the present findings. First, the sample size was relatively small, and further studies are needed with more subjects. Although the fingerprinting approach is designed to extract subject-specific information, larger samples are needed to confirm the generalizability of our results. Second, gait recordings were obtained in a single session, limiting our ability to assess the longer-term stability of individual coordination patterns. Longitudinal data is important to determine whether the observed reductions in motor individuality in older adults are progressive or stable over time. Despite these limitations, this study provides novel insights into how aging affects the individuality and structure of gait coordination, with several promising implications. First, the use of kinectome-based fingerprinting may offer a sensitive tool for monitoring motor aging in both research and clinical settings. In addition, the gender imbalance in our sample, with a prevalence of male participants, represents a further limitation that may have influenced the results. Future studies with more balanced samples are therefore essential to clarify the extent to which gender differences contribute to the patterns of gait individuality and stability observed here. Second, the observed reduction in motor distinctiveness among older adults suggests the potential of this method for detecting early signs of increased rigidity or reduced adaptability, which are factors often associated with fall risk and motor decline. Third, inspired by principles from network theory, our method employs an edge-centric approach that shifts the focus from isolated joint activity to the dynamic interplay between joint pairs in a subject-specific perspective [44]. Future studies should also take into consideration different motor tasks or regular sport and physical activity practice, as this may have an effect on different aspects of an individual, such as motor, cognitive and emotional abilities [45,46,47]. Furthermore, analyzing coordination mechanisms at the muscular level could further contribute to current knowledge and help determine whether similar findings are observed at the neuromuscular level [48,49].

In conclusion, our findings highlight that, while intra-individual consistency of gait coordination remains preserved with age, the individual distinctiveness of motor patterns is reduced in older adults. This suggests that aging not only alters motor function but also reshapes the structural identity of movement. By applying a kinectome-based fingerprinting framework, we demonstrate the utility of network-level analysis in capturing age-related shifts in motor coordination that are not evident through traditional gait metrics. These results may prove useful for future applications of this method in investigating motor aging, identifying early signs of functional decline, and supporting more personalized approaches to motor assessment and rehabilitation.

## Figures and Tables

**Table 1:** Characteristics of the study participants.

Variable	Adults (n = 12)	Older Adults (n = 14)
Age, mean ± SD (years)	41.6 ± 6.4	73.5 ± 3.5
Sex (M/F)	10/2	10/4
Height, mean ± SD (cm)	174.2 ± 7.8	168.1 ± 6.9
Weight, mean ± SD (kg)	73.4 ± 9.1	70.6 ± 8.3
BMI, mean ± SD	24.2 ± 2.4	24.9 ± 2.6
Foot length, mean ± SD (cm)	26.7 ± 1.0	25.6 ± 1.0
Daily steps, mean ± SD	8100 ± 1400	6200 ± 1200
Regular physical activity (%) *	58	43
Joint flexibility (ankle dorsiflexion, degrees) **	17.4 ± 3.1	14.0 ± 3.4
Neurological disorders (e.g., Parkinson’s)	0	0
Musculoskeletal impairments (%)	0	0
Chronic cerebrovascular disease (%)	0	0
History of orthopedic surgery (%)	0	0
Vestibular/inner ear pathology (%)	0	0
Current pharmacological therapy (%)	17 (mild hypertension)	29 (antihypertensives, statins)
Alcohol consumption (% occasional)	33	29

* Defined as ≥150 min/week of moderate physical activity according to WHO guidelines. ** Measured as maximum passive ankle dorsiflexion with extended knee, in degrees.

**Table 2:** Group comparisons of gait fingerprinting metrics. Reported statistics include *p*-values, Cohen’s d, and η^2^.

Variable	Adults (M ± SD)	Older Adults (M ± SD)	*p*-Value	Cohen’s d	Interpretation	η^2^
Iself	0.93 ± 0.05	0.92 ± 0.06	0.628	0.18	Very small effect	–
Iothers	0.74 ± 0.05	0.82 ± 0.04	<0.001	−1.78	Very large effect	–
DR	0.97 ± 0.07	0.85 ± 0.19	0.040	0.81	Large effect	–
ANOVA (Iothers/Iextra)	–	–	0.006	–	–	0.15 (Large)

## Data Availability

The data presented in this study are available on request from the corresponding author. The data are not publicly available due to privacy restrictions.
